# Supplementary material for: Local synaptic inputs support opposing, network-specific odor representations in a widely projecting modulatory neuron
Source: eLife. 2019 Jul 2;8:e46839. doi: 10.7554/eLife.46839 (PMC6660217; doi:10.7554/eLife.46839)
Supplement: Supplementary file 1. [file elife-46839-supp1.docx]

| **CSDn Compartmental Model** | | | |
| --- | --- | --- | --- |
| **Parameter** | **Value Range** | **Units** | **Description** |
| **R_a_** | 0.0001-5000 | Ωcm | Axial Resistance |
| **C_m_** | 0.1-2 | μF/cm^2^ | Membrane Capacitance |
| **g_leak_** | 10^-6^-0.1 | S/cm^2^ | Membrane Leak Conductance |
| **t** | 0.025 | ms | Sampling Time Step |
